# Are We Missing the Target When Measuring Quality of Life?

**DOI:** 10.1093/geroni/igaa057.1201

**Published:** 2020-12-16

**Authors:** Leah Janssen, Kate de Medeiros

**Affiliations:** 1 Miami University, Hamilton, Ohio, United States; 2 Miami University, Oxford, Ohio, United States

## Abstract

Over the last several decades, many instruments have been created to measure quality of life (QoL) in older adults, particularly for intervention research on individuals living with dementia. However, since definitions of QoL lack standardization across the research literature, the question of how to holistically capture an elusive and expansive concept such as QoL remain. This research uses qualitative content analysis to explore definitions and domains of QoL with an eye toward overlap and gaps. Definitions of QoL were extracted from gerontology encyclopedia entries and other peer-reviewed supplemental resources and analyzed for themes using Dedoose qualitative software. Results revealed three over-arching themes: no standardized or universal definition of QoL, use of subjective and objective factors for measurement, and varying domains of QoL. Additionally, we further distilled theme three to identify eight unique QoL domains: 1) economic/financial, 2) environment, 3) ADL/IADL function, 4) participation in activities, 5) personal resources, 6) physical health, 7) psychological well-being, and 8) social/relational, the total of which were only found in one of 15 definitions of QoL. Overall, findings led to an overarching definition of QoL that cuts across multiple dimensions and factors. We argue that by having all eight domains our understanding and measurement of QoL is enhanced, thereby improving our assessment of existing definitions of QoL, as well as the instruments used to measure QoL.

